# Supplementary material for: Differential Contribution of Protein Factors and 70S Ribosome to Elongation
Source: Int J Mol Sci. 2021 Sep 5;22(17):9614. doi: 10.3390/ijms22179614 (PMC8431766; doi:10.3390/ijms22179614)
Supplement: Supplementary file 1 [file ijms-22-09614-s001.zip › ijms-1351055-supplementary.pdf]

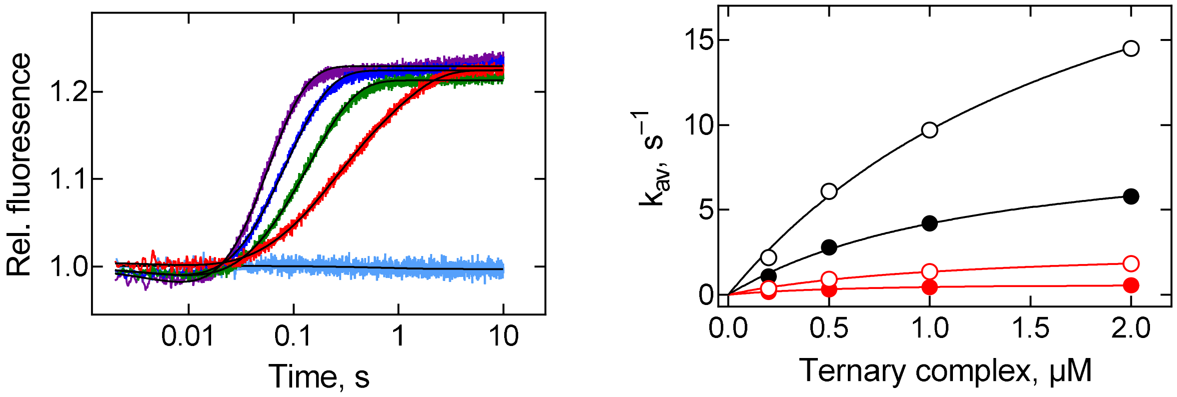

(**a**) (**b**)

Figure S1. A-site binding kinetics monitored by fluorescence label BODIPY FL at methionine moiety of tRNA. The ribosomal complexes containing BPY-Met-$tRNA^{Met}$ in the P site were rapidly mixed with increasing amount of ternary complex, EF-Tu·GTP·Phe-$tRNA^{Phe}$. (a) Fluorescence time courses of BPY-labelled ribosomal complex (0.1 μM) interaction with ternary complex (0.2 μM, red; 0.5 μM, green; 1 μM, dark blue; 2 μM, violet), containing EF-Tu from E. coli at 37 °C or buffer $TAKM_7$ (blue). Each time course reflects the average of 5–7 technical replicates. Black lines show double-exponential fits. (b) Concentration dependence of average rate constants (k_{av}) on ternary complex (0.2–2 μM). k_{av} values were estimated by double-exponential fitting of time courses as in (a) for A-site reactions with EF-Tu from E. coli at 20 °C (black circles), at 37 °C (black open circles), EF-Tu from T. thermophilus at 20 °C (red circles), at 37 °C (red open circles). Lines show hyperbolic fits, numerical values are given in the text. Error bars (s.e.m.) are calculated by the GraphPad Prism software from at least two independent experiments with 5–7 technical replicates for each concentration, however, do not exceed the size of symbols.
